# Clinical Insights Into Default Mode Network Abnormalities in Mild Traumatic Brain Injury: Unraveling Axonal Injury Through Functional, Structural, and Molecular Analyses

**DOI:** 10.1111/cns.70188

**Published:** 2024-12-26

**Authors:** Dewen Ru, Jun Zhang, Lichao Wei, Zengyu Zhang, Yue Wang, Fengyuan Zhou, Gang Wu, Qiang Yuan, Zhuoying Du, Ersong Wang, Jin Hu

**Affiliations:** ^1^ Department of Neurosurgery, Huashan Hospital, Shanghai Medical College Fudan University Shanghai China; ^2^ Department of Neurosurgery, Jinshan Hospital Fudan University Shanghai China; ^3^ National Center for Neurological Disorders Shanghai China; ^4^ Shanghai Key Laboratory of Brain Function and Restoration and Neural Regeneration Shanghai China; ^5^ Neurosurgical Institute of Fudan University Shanghai China; ^6^ Shanghai Clinical Medical Center of Neurosurgery Shanghai China; ^7^ Department of Neurosurgery Xinhua Hospital Affiliated to Shanghai Jiao Tong University School of Medicine Shanghai China; ^8^ Department of Neurology, Minhang Hospital Fudan University Shanghai China

**Keywords:** axonal injury, default mode network, diffusion tensor imaging, functional connectivity, mild traumatic brain injury

## Abstract

**Background:**

Mild traumatic brain injury (mTBI) frequently results in persistent cognitive, emotional, and functional impairments, closely linked to disruptions in the default mode network (DMN). Understanding the mechanisms driving these network abnormalities is critical for advancing diagnostic and therapeutic strategies.

**Methods:**

This study adopted a multimodal approach, combining functional connectivity (FC) analysis, diffusion tensor imaging (DTI), and gene expression profiling to investigate DMN disruptions in mTBI. A primary focus was placed on the middle cingulate cortex (MCC), a region consistently identified with increased connectivity. We explored the structural and molecular changes underlying this phenomenon. Receiver operating characteristic (ROC) curve analysis was utilized to assess the diagnostic potential of DTI‐derived metrics, while white matter tractography was employed to explore structural connectivity between the MCC and the dorsolateral prefrontal cortex (DLPFC).

**Results:**

Our findings revealed significant disruptions in DMN connectivity, with the MCC prominently involved in mTBI pathology. DTI analyses identified pronounced axonal injury in the MCC, characterized by decreased fractional anisotropy (FA) and axial diffusivity (AD), alongside increased isotropy (ISO), indicating compromised white matter integrity and diffuse axonal injury. Gene expression profiling revealed the upregulation of pathways related to synaptic transmission, ion channel regulation, and axonal injury response. ROC analysis demonstrated that ISO serves as a particularly effective biomarker for mTBI, showing high diagnostic accuracy (AUC = 0.871). White matter tractography further confirmed strong structural connectivity between the MCC and the DLPFC, identifying potential therapeutic targets for neuromodulation.

**Conclusion:**

This study provides robust evidence that diffuse axonal injury plays a pivotal role in DMN abnormalities observed in mTBI. The integration of FC, DTI, and gene expression profiling offers a comprehensive framework for understanding mTBI's impact on brain networks. Our findings also highlight the DLPFC as a promising target for therapeutic interventions aimed at addressing cognitive and emotional deficits associated with mTBI.

## Graphical abstract

This study explores the relationship between mild traumatic brain injury (mTBI) and disruptions in the default mode network (DMN) using a multimodal approach. Through functional connectivity analysis, diffusion tensor imaging, and gene expression profiling, the research highlights the role of diffuse axonal injury in DMN abnormalities, particularly in the middle cingulate cortex (MCC). The study suggests potential therapeutic targets for neuromodulation, such as the dorsolateral prefrontal cortex (DLPFC), to mitigate cognitive and emotional disturbances associated with mTBI.

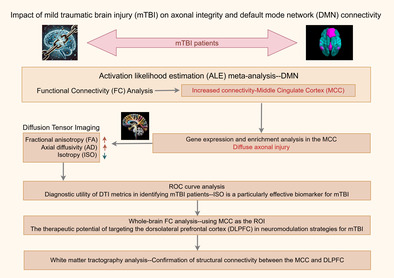

## 1 Introduction

Mild traumatic brain injury (mTBI) is a prevalent yet frequently underdiagnosed condition, affecting millions globally and leading to a wide range of cognitive, emotional, and behavioral outcomes [1]. While the majority of individuals recover within weeks, a significant subset experiences persistent symptoms, collectively referred to as post‐concussion syndrome (PCS), which can substantially diminish the quality of life [2]. Emerging neuroimaging research has underscored the pivotal role of the default mode network (DMN)—a critical network associated with self‐referential thinking and memory functions—in the pathophysiology of mTBI. Studies have consistently demonstrated that mTBI disrupts DMN connectivity, with these disruptions correlating with both cognitive impairments and symptom severity [3, 4]. Therefore, investigating DMN abnormalities is crucial for elucidating the neural mechanisms underlying persistent symptoms and for guiding the development of targeted therapeutic interventions [5].

Diffuse axonal injury, a hallmark of mTBI, is characterized by damage to white matter tracts and has been shown to significantly disrupt both structural and functional connectivity (FC) within various brain networks [6]. Despite this understanding, the specific relationship between axonal damage and dysfunction in the DMN remains insufficiently explored. To bridge this knowledge gap, a more comprehensive investigation is needed, integrating multimodal approaches that combine FC analysis, diffusion tensor imaging (DTI), and molecular profiling [7, 8]. In this study, we employ such an integrative approach to examine the structural, functional, and molecular mechanisms underlying DMN abnormalities in mTBI, with a particular focus on the middle cingulate cortex (MCC), a region frequently implicated in connectivity alterations following mTBI [9, 10]. Given the dorsolateral prefrontal cortex's (DLPFC) crucial role in cognitive functions such as working memory, processing speed, and executive control, and its robust connectivity with the MCC and anterior cingulate cortex (ACC), disruptions in DLPFC connectivity may contribute significantly to the cognitive impairments observed in mTBI and other neurological conditions [11].

This study investigates abnormalities in the DMN in mTBI using a multimodal approach that integrates FC analysis, DTI, and gene expression profiling. Our findings reveal significant disruptions in DMN connectivity, particularly within the MCC. Gene expression analysis identified the upregulation of pathways associated with synaptic transmission and axonal injury. DTI results further demonstrated diffuse axonal injury, characterized by reduced fractional anisotropy (FA) and increased isotropy (ISO). These findings indicate that axonal damage is a central contributor to DMN dysfunction, highlighting the DLPFC as a key therapeutic target for mTBI interventions.

## 2 Methods

### 2.1 Search Strategies and Selection Criteria

#### 2.1.1 Literature Search Strategy

This study adheres to the guidelines outlined in the Preferred Reporting Items for Systematic Reviews and Meta‐Analyses (PRISMA) statement [12]. Two authors independently conducted a comprehensive literature search in the PubMed database using the keywords “mild traumatic brain injury” or “concussion” and “magnetic resonance imaging” or “MRI” or “functional magnetic resonance imaging” or “fMRI” or “functional connectivity” or “FC” or “brain network” or “default mode network” or “DMN.” The search covered publications up to October 2023. Duplicate records and irrelevant studies were excluded based on a screening of titles, abstracts, and, when necessary, full‐text reviews.

#### 2.1.2 Literature Selection

**FIGURE 1:**
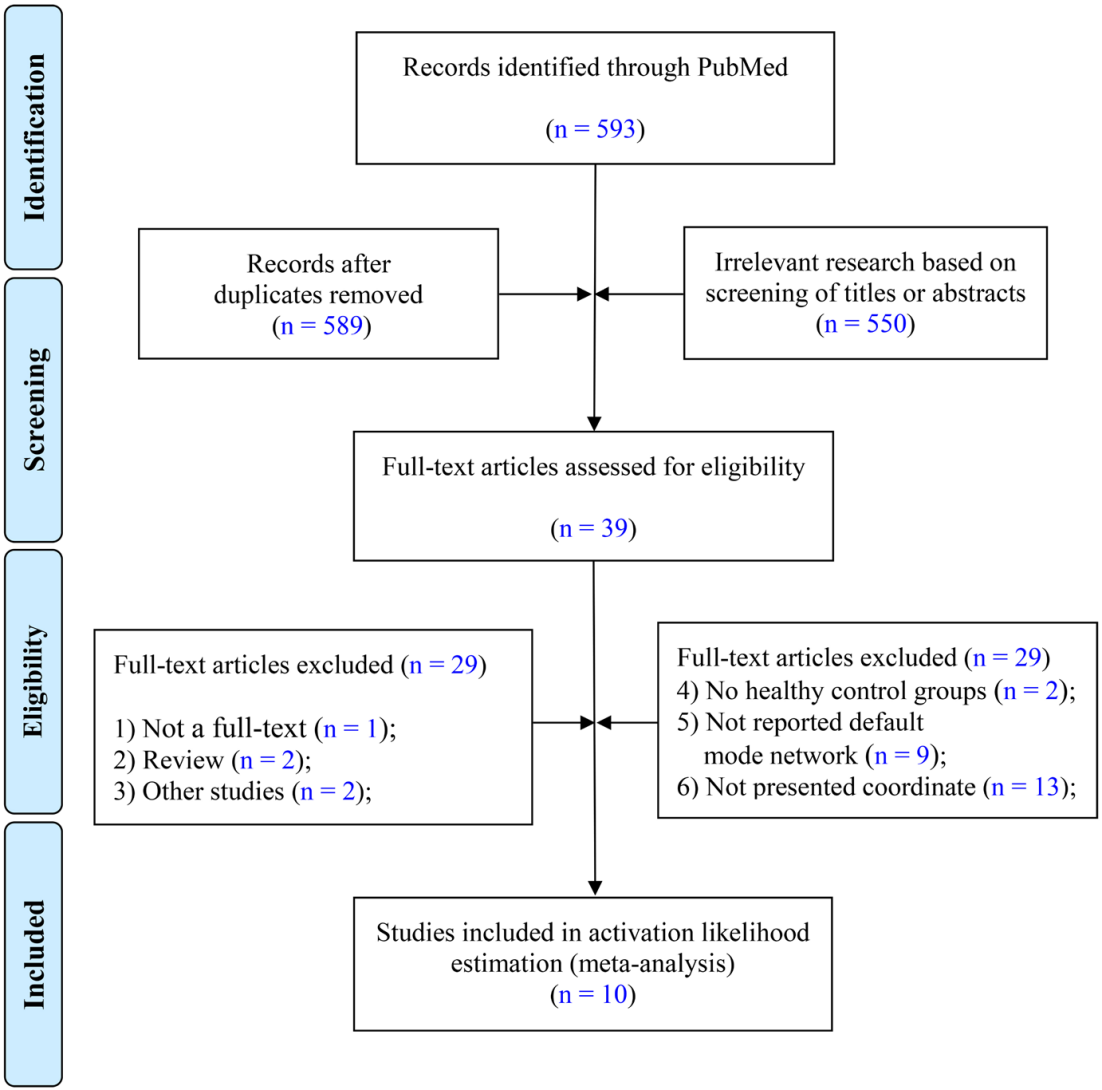
Flowchart of studies selection process for meta‐analysis on DMN abnormalities in mTBI patients. As is shown above, 10 studies met all inclusion criteria and were incorporated into the final ALE meta‐analysis, which aimed to investigate DMN abnormalities associated with mTBI. The use of these criteria ensured a rigorous and focused analysis of the literature on DMN alterations in the context of mTBI. ALE, activation likelihood estimation; DMN, default mode network; mTBI, mild traumatic brain injury.

The research focused on the Default Mode Network (DMN) and its FC architecture, specifically reporting the spatial coordinates of abnormal brain regions. Participants were required to meet the diagnostic criteria for mTBI as defined by established guidelines [13, 14]. Figure 1 outlines the study selection process for investigating DMN abnormalities in mTBI patients. The initial PubMed search yielded 593 potential studies. After removing duplicates, 589 articles remained and were screened based on their titles and abstracts, resulting in the exclusion of 550 studies that did not meet the relevance criteria. Next, 39 full‐text articles were assessed, with 29 studies excluded due to reasons such as lack of full‐text availability, being review articles, misalignment with research objectives, absence of healthy control groups, failure to report on the DMN, or not providing spatial coordinates. Ultimately, 10 studies met the stringent inclusion criteria and were included in the final ALE analysis.

#### 2.1.3 Data Extraction and Quality Assessment

Two authors independently extracted relevant data from studies eligible for the meta‐analysis using predefined forms and text. The extracted data were then cross‐checked by the authors to ensure accuracy. The extracted variables included the first author's name, publication year, study type, sample size, age, number of participants in the mTBI and control groups, gender ratio, MRI parameters, acquisition time, and brain networks or regions of interest (ROI). The quality of the included studies was assessed using the Newcastle‐Ottawa Scale (NOS) [15], with a maximum score of 9. Studies scoring above 6 were classified as high‐quality, while those scoring below were considered low‐quality (An asterisk was used to denote this classification in the analysis). To maintain consistency, regular meetings were held among analysts to review results and resolve discrepancies through collective discussion, thereby minimizing subjective bias and ensuring uniform application of the standardized analysis protocol.

### 2.2 Activation Likelihood Estimation (ALE)

We employed the ALE algorithm to conduct quantitative, coordinate‐based meta‐analyses of functional neuroimaging results. This method assesses the convergence of activation foci across multiple experiments, comparing these foci to a randomly distributed set of coordinates. A key advantage of the ALE method is its ability to utilize the coordinate lists commonly reported in neuroimaging studies as input data. Prior to the main analysis, coordinates reported in Talairach space were converted to Montreal Neurological Institute (MNI) standard space using the GingerALE conversion tool (icbm2tal transformation) [16]. The converted coordinates were formatted according to the recommendations of the ALE guidebook and analyzed using BrainMap GingerALE 3.0.2 (http://www.brainmap.org/ale), which simulates stochastic coordinates based on study size to account for noise and enhance the robustness of the results.

Briefly, the ALE algorithm involves four key steps: modeling the activation map, calculating voxel values, constructing a null distribution, and identifying true activation values that surpass a statistical threshold. In this study, the specific parameters were set as follows: full‐width half‐maximum (FWHM) at 6 mm and the family‐wise error (FWE) correction with a cluster‐level threshold of *p* < 0.05, alongside a voxel‐level threshold of *p* < 0.01 (100 permutations). Using ALE, we searched for brain regions exhibiting activation or suppression in three comparisons: (i) mTBI within 7 days post‐injury versus healthy controls, (ii) mTBI more than 7 days post‐injury versus healthy controls, and (iii) all mTBI cases versus healthy controls.

### 2.3 Gene Expression and Enrichment Analysis

After initial quality control and normalization of the microarray data, preprocessing was conducted with reference to the Allen Brain Atlas (http://help.brain‐map.org/display/humanbrain/documentation/) [17] This process included probe‐to‐gene annotation validation, probe cleaning, and selection. To address the issue of multiple probes corresponding to individual genes, we utilized the “collapserws” function from the “WGCNA” package, selecting probes with the highest average expression levels to represent the corresponding genes. A total of 20,738 gene expression profiles and 3695 matched brain tissue samples were obtained. The genetic data were normalized at the overall level to account for group‐level effects while minimizing individual variation. For each of the 3695 tissue samples, the MCC was mapped using its MNI center‐of‐mass coordinates as the origin. Samples covering more than 50% of the whole‐brain voxels were defined as valid samples. These samples were then mapped to MCC brain regions to identify the region with the maximum voxel overlap, and their average expression levels were calculated.

Subsequently, the preprocessed data were mapped onto the 2D Conte69 template using the Human Connectome Workbench (https://humanconnectome.org/software/connectome‐workbench). Gene expression differences between the target regions and random samples were compared using a permutation test (1000 replicates, *p* < 0.01). Next, we conducted an enrichment analysis of the up‐ or down‐regulated genes using the Metascape platform (https://metascape.org/gp/index.html) [18]. The steps included: (i) identifying gene identifiers and converting them to Entrez gene IDs, (ii) annotating the genes with information on function, type, description, and protein classification, (iii) clustering the gene sets, (iv) performing enrichment analysis using the full Metascape database, and (v) generating an enrichment graphical report.

### 2.4 Participants

Patients with mTBI who attended the Department of Neurosurgery at Huashan Hospital between October 2022 and June 2024 were prospectively enrolled in this study. The study was approved by the Ethics Committee of Huashan Hospital (Approval number: KY2022‐062), and informed consent was obtained from all patients or their families.

Inclusion criteria: (i) Participants aged between 18 and 80 years were included. (ii) Participants had to meet the diagnostic criteria for mTBI as outlined by established guidelines [13, 14], which included: Loss of consciousness: Duration of less than 30 min; Post‐traumatic amnesia: Duration of less than 24 h; Glasgow Coma Scale (GCS): A GCS score between 13 and 15, measured 1 h after the injury. (iii) All participants were right‐handed to control for potential hemispheric dominance effects on brain imaging.

Exclusion criteria: Participants were excluded if they met any of the following conditions: (i) Evidence of intracranial hemorrhage or other structural brain injuries on imaging; (ii) A history of neurological disorders (e.g., epilepsy, stroke) or psychiatric conditions that could confound the results; (iii) Contraindications to MRI, such as metallic implants or claustrophobia.

Additionally, 40 healthy controls were randomly selected from our team's brain imaging database for FC and structural connectivity analyses. These inclusion criteria were carefully designed to ensure a homogenous study population, specifically focusing on individuals with mild traumatic brain injury while minimizing potential confounding factors. There were no restrictions based on sex or education level.

### 2.5 Resting‐State fMRI (Rs‐fMRI) Scanning

Whole‐brain imaging was performed using a GE Discovery MR750 3 Tesla MRI scanner equipped with an 8‐channel head coil. Functional images were acquired using a gradient echo sequence with the following parameters: repetition time (TR) = 2000 ms, echo time (TE) = 30 ms, field of view (FOV) = 240 × 240 mm, flip angle (FA) = 70°, matrix size = 64 × 64, slice thickness = 3.5 mm, and 33 slices. A total of 240 brain volumes were collected. For structural imaging, 3D T1‐weighted imaging (T1WI) was conducted using a rapid equivalent voxel cranial thin‐layer sequence with the following parameters: TR = 8.16 ms, TE = 3.18 ms, FA = 12°, matrix size = 256 × 256, slice thickness = 1.0 mm, and 168 slices.

#### 2.5.1 Rs‐fMRI Pre‐Processing

Image preprocessing was performed using Data Processing & Analysis of Brain Imaging (DPABI) v7.0 (http://rfmri.org/dpabi) based on the MATLAB 2016b (https://www.mathworks.com) platform. The data processing steps were as follows: (i) DICOM data were converted to NIFTI format. (ii) The first 10 volumes of functional time points were discarded to allow participants to adapt to the scanner noise. (iii) The 33rd slice was used as a reference for temporal correction. (iv) The Friston 24‐parameter model was applied for head motion correction, and subjects with more than 2 mm of translation or greater than 2° of rotation were excluded. (v) Registration of structural and functional images was performed to prepare for subsequent segmentation and normalization. (vi) Segmentation and spatial normalization were conducted using the DARTEL (Diffeomorphic Anatomical Registration Through Exponentiated Lie) algorithm. Before spatial normalization, covariate regression was performed to remove nuisance signals, including global signal, Friston 24 head motion parameters, white matter signal, and cerebrospinal fluid signal. (vii) Linear detrending was applied. (viii) A band‐pass filter (0.01–0.1 Hz) was used. (ix) Spatial smoothing was performed using a 3 × 3 × 3 mm^3^ isotropic Gaussian kernel to reduce noise in the normalized functional images.

#### 2.5.2 FC Analysis

FC analyses were performed using DPABI v7.0. Briefly, the MCC was selected as ROI, and the mean time series of blood oxygenation level‐dependent (BOLD) signals from the ROI were used as the reference time course. Spearman correlation coefficients were calculated to assess the cross‐correlation between the mean signal changes in the ROI and the time series of voxels in other brain regions. To improve the normality of the correlation coefficients, a Fisher *z*‐transformation was applied, converting *r*‐valued matrices to *z*‐matrices. A one‐sample *t*‐test was then used to construct a whole‐brain FC atlas for the MCC.

### 2.6 MCC Functional Decoding

Additionally, Neurosynth (https://www.neurosynth.org/) was utilized for functional decoding analyses of brain regions. Neurosynth provides voxel‐level functional terminology and task decoding, incorporating a large number of task activation maps. This platform automatically integrates analyses and decoding functions based on a comprehensive dataset of over 11,000 published neuroimaging studies. The goal of this analysis was to identify the functional terms associated with the MCC brain regions.

### 2.7 DTI Scanning and Tractography

Diffusion tensor imaging (DTI) data were acquired using a single‐shot echo‐planar imaging sequence with the following parameters: TR = 5300 ms, TE = 82 ms, field of view (FOV) = 256 × 256 mm, flip angle (FA) = 90°, matrix size = 128 × 128, slice thickness = 3 mm, no slice gap, and 40 slices. The dataset included four b‐0 fields and 64 diffusion gradients along noncollinear orientations (*b*‐value = 1000 s/mm^2^).

The data were further analyzed using FSL (https://fsl.fmrib.ox.ac.uk/fsl/fslwiki/FSL) and DSI Studio (https://dsi‐studio.labsolver.org/) software. The analysis followed these steps: (i) DICOM data were converted to NIFTI format using dcm2niix. (ii) Diffusion‐weighted images were manually checked for quality, and subjects with poor‐quality scans were excluded. (iii) An SRC file was created by loading the data and adding the corresponding *b*‐values (*b*val) and *b*‐vectors (*b*vec). (iv) Masks were generated using thresholding, scaling, smoothing, and fragmentation filtering techniques. (v) TOPUP and EDDY algorithms from FSL were used for motion and distortion correction. (vi) The data were reconstructed into Montreal Neurological Institute (MNI) space using the Q‐quality algorithm.

FA, axial diffusivity (AD), and ISO values were extracted from the MCC using the Statistics module. Finally, a structural connectivity loop was constructed using the right DLPFC and the MCC as ROIs.

### 2.8 mTBI Identification

Receiver operating characteristic (ROC) curve analysis was conducted to evaluate the diagnostic efficacy of DTI metrics—FA, AD, and ISO—in distinguishing between patients with mTBI and healthy controls. The area under the curve (AUC), sensitivity, and specificity were calculated for each metric. Optimal cut‐off values were determined using the Youden index. The goal of this study was to identify highly sensitive imaging biomarkers that could be applied in clinical practice for mTBI diagnosis, particularly in cases where conventional imaging techniques fail to reveal obvious abnormalities.

### 2.9 Statistical Analysis

All statistical analyses were performed using SPSS version 26 (https://www.ibm.com/products/spss‐statistics) and MedCalc (https://www.medcalc.org/) software. The Shapiro–Wilk test was used to assess whether the data followed a normal distribution. If the *p* value exceeded the significance level (0.05), we did not reject the null hypothesis, indicating that the data were approximately normally distributed. For datasets that did not meet the criteria for normality, non‐parametric tests were employed. Group comparisons were conducted using two‐sample *t*‐tests or non‐parametric tests, depending on the distribution of the data. A significance level of *p* < 0.05 was applied to all statistical tests.

## 3 Results

### 3.1 Characteristics and Quality Assessment of Included Studies

Table 1 presents a comprehensive summary of the characteristics and quality assessments of the studies included in the meta‐analysis [4, 7, 19, 20, 21, 22, 23, 24, 25, 26]. While the studies shared several commonalities, such as design, participant demographics, MRI specifications, and post‐injury acquisition times, they also exhibited unique aspects in terms of the specific brain networks or ROIs examined. The quality of each study was evaluated using the Newcastle‐Ottawa Scale (NOS), which assesses selection, comparability, and outcome measures, ensuring the robustness of the included studies. The table provides the quality ratings, showing that most studies achieved high scores on the NOS, reflecting methodological rigor. Additionally, it highlights key study aspects such as sample size, participant age, diagnostic criteria for mTBI, and MRI parameters used. These details offer important context for interpreting the meta‐analysis results.

**TABLE 1:** Characteristics and quality of the included studies.

Study and year	Design	Age	Number	MRI	Acquisition time (post‐injury)	Brain networks or ROI	Quality
		mTBI, control	mTBI, control				
Sheth, 2021	CSS	20–54	49, 25	3T	> 3 months	DMN	★★★★★★
Amir, 2021	CSS	18–60	27, 26	3T	1 month	DMN, TPN, SN, SMN, VN	★★★★★★★
D'Souza, 2020	LS	30.40 ± 10.34, 30.82 ± 7.39	60, 60	3T	Within 7 days	DMN, ECN, SMN, AN	★★★★★★★
Niu, 2018	LS	34.7 ± 12.2, 34.9 ± 11.6	70, 46	3T	Within 7 days	DMN	★★★★★★
Murdaugh, 2018	LS	14–18	16, 12	3T	Within 7 days	DMN	★★★★★★★
Vergara, 2017	CSS	27.3 ± 9.0, Matched	47, 47	3T	Within 7 days	Most RSNs	★★★★★
Nathan, 2015	CSS	25.6 ± 4.4, 26.4 ± 5.8	15, 12	3T	2–10 months	DMN	★★★★★
Sours, 2015	LS	38.9 ± 15.9, 39.3 ± 17.2	28, 28	3T	137–266 days	DMN, TPN	★★★★★★
Zhou, 2012	CSS	37.8 ± 12.9, 32.6 ± 10.0	23, 18	3T	3–53 days	DMN	★★★★★★
Stevens, 2012	CSS	31.7 ± 13.9, 28.9 ± 9.92	30, 30	3T	13–136 days	Most RSNs	★★★★★★★

Abbreviations: AN, auditory network; CSS, cross‐sectional study; DMN, default mode network; ECN, executive control network; LS, longitudinal study; M, male; MRI, magnetic resonance imaging; mTBI, mild traumatic brain injury; ROI, region of interest; RSNs, resting state networks; SMN, sensorimotor network; SN, salience network; T, Tesla; TPN, task positive networks; VN, visual network.

### 3.2 Dual‐Phase Alterations in DMN Connectivity Following mTBI: Acute Disruptions and Compensatory Mechanisms Beyond 7 Days Post‐Injury

**FIGURE 2:**
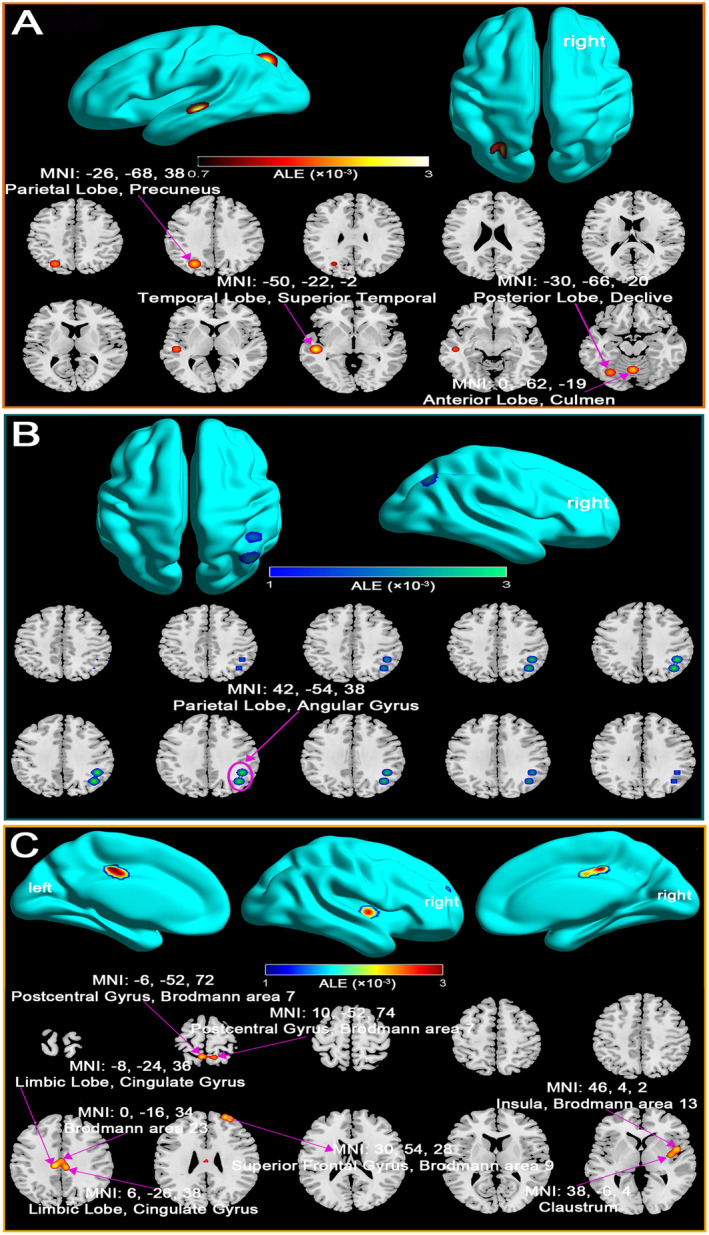
Changes in DMN connectivity in mTBI patients compared to healthy controls across acute and subacute phases. Acute Phase (within 7 days post‐injury): (A) Increased DMN connectivity. 3D surface renderings of the brain highlight regions where mTBI patients exhibited significantly increased DMN connectivity relative to healthy controls. Key regions include the Posterior Lobe, Declive, Temporal Lobe, Superior Temporal Gyrus, Parietal Lobe, Precuneus, Anterior Lobe, and Culmen. (B) Decreased DMN connectivity. 3D brain renderings show regions where mTBI patients exhibited significantly decreased DMN connectivity compared to healthy controls. Key regions include the Parietal Lobe and Angular Gyrus. Subacute Phase (beyond 7 days post‐injury): (C) The 3D surface renderings depict regions in both the left and right hemispheres where mTBI patients exhibited significantly increased DMN connectivity. Key regions include the Limbic Lobe, Cingulate Gyrus, Brodmann area 23, Insula, Brodmann area 13, Claustrum, Postcentral Gyrus, Brodmann area 7, Superior Frontal Gyrus, and Brodmann area 9. ALE, activation likelihood estimation; MNI, Montreal Neurological Institute.

Our analysis reveals distinct alterations in Default Mode Network (DMN) connectivity in mTBI patients across two post‐injury phases. In the acute phase (within 7 days), mTBI patients demonstrated both increased and decreased DMN connectivity compared to healthy controls. Increased connectivity was observed in regions such as the posterior lobe, precuneus, and superior temporal gyrus, suggesting a compensatory response aimed at maintaining cognitive functions despite network disruption (Figure 2A; Table S1). In contrast, decreased connectivity in areas like the parietal lobe and angular gyrus likely reflects the immediate effects of axonal injury and impaired network integration (Figure 2B; Table S1).

Beyond 7 days post‐injury, the analysis indicated predominantly elevated DMN connectivity, especially in key regions including the cingulate gyrus, insula, and superior frontal gyrus (Figure 2C; Table S2). This increased connectivity may reflect ongoing compensatory neural mechanisms, potentially driven by neural plasticity in response to axonal damage. Notably, no regions of decreased connectivity were identified during this subacute phase, emphasizing the brain's adaptive responses to preserve cognitive function.

These findings highlight the importance of early intervention during the acute phase and suggest that bolstering compensatory processes in the later phases may help mitigate long‐term cognitive deficits associated with mTBI.

### 3.3 Complex Alterations in DMN Connectivity in mTBI Patients: Evidence of Both Compensatory and Disruptive Network Changes

**FIGURE 3:**
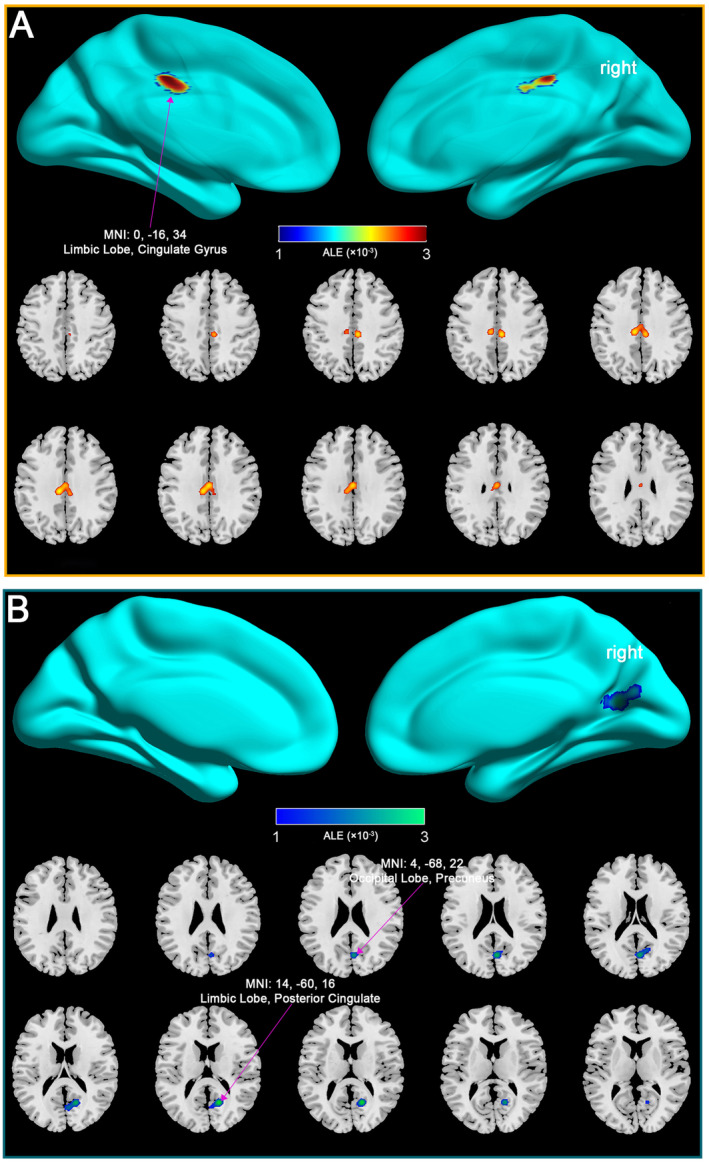
Increased DMN connectivity in all mTBI patients compared to healthy controls. (A) Increased DMN connectivity: The top row presents 3D surface renderings of the brain, highlighting regions in both hemispheres where mTBI patients exhibited significantly increased DMN connectivity. Key regions include the Limbic Lobe and Cingulate Gyrus. The bottom row shows axial slices at specific *z*‐coordinates (*z* = 43, *z* = 35.5), offering a more detailed view of the areas with enhanced connectivity. (B) Decreased DMN connectivity: This panel displays regions where mTBI patients showed significantly decreased DMN connectivity compared to healthy controls. Key regions include the Limbic Lobe, Posterior Cingulate, Occipital Lobe, and Precuneus. The top row provides 3D brain renderings, while the bottom row presents axial slices at various *z*‐coordinates (*z* = 28, *z* = 18) to illustrate these areas with decreased connectivity. ALE, activation likelihood estimation; MNI, Montreal Neurological Institute.

The ALE meta‐analysis, which included all mTBI patients regardless of time since injury, revealed significant and complex alterations in DMN connectivity compared to healthy controls (Table S3). As shown in Figure 3A, the analysis identified several key regions within the DMN, including the limbic lobe and cingulate gyrus, where mTBI patients exhibited significantly increased connectivity. These regions are critical components of the DMN, involved in self‐referential processing, memory retrieval, and maintaining a resting brain state. The observed increase in connectivity in these areas likely represents compensatory neural mechanisms, wherein the brain adapts to injury by enhancing functional connectivity (FC) in critical regions to preserve cognitive function.

Conversely, Figure 3B illustrates regions within the DMN where mTBI patients demonstrated decreased connectivity, particularly in the limbic lobe, posterior cingulate, occipital lobe, and precuneus. These reductions in connectivity suggest impaired network integration and cognitive processing, which are commonly associated with the cognitive deficits and persistent symptoms observed in mTBI patients.

Overall, the findings from this comprehensive analysis suggest that mTBI induces a complex pattern of both increased and decreased DMN connectivity. These changes reflect the brain's dual response to injury—engaging compensatory mechanisms in some regions while experiencing disruption and loss of function in others. Understanding these opposing dynamics is essential for developing effective interventions that address the full spectrum of mTBI's impact on brain function.

### 3.4 Molecular and Structural Mechanisms of MCC Alterations in mTBI: Compensatory Connectivity and Evidence of Diffuse Axonal Injury

**FIGURE 4:**
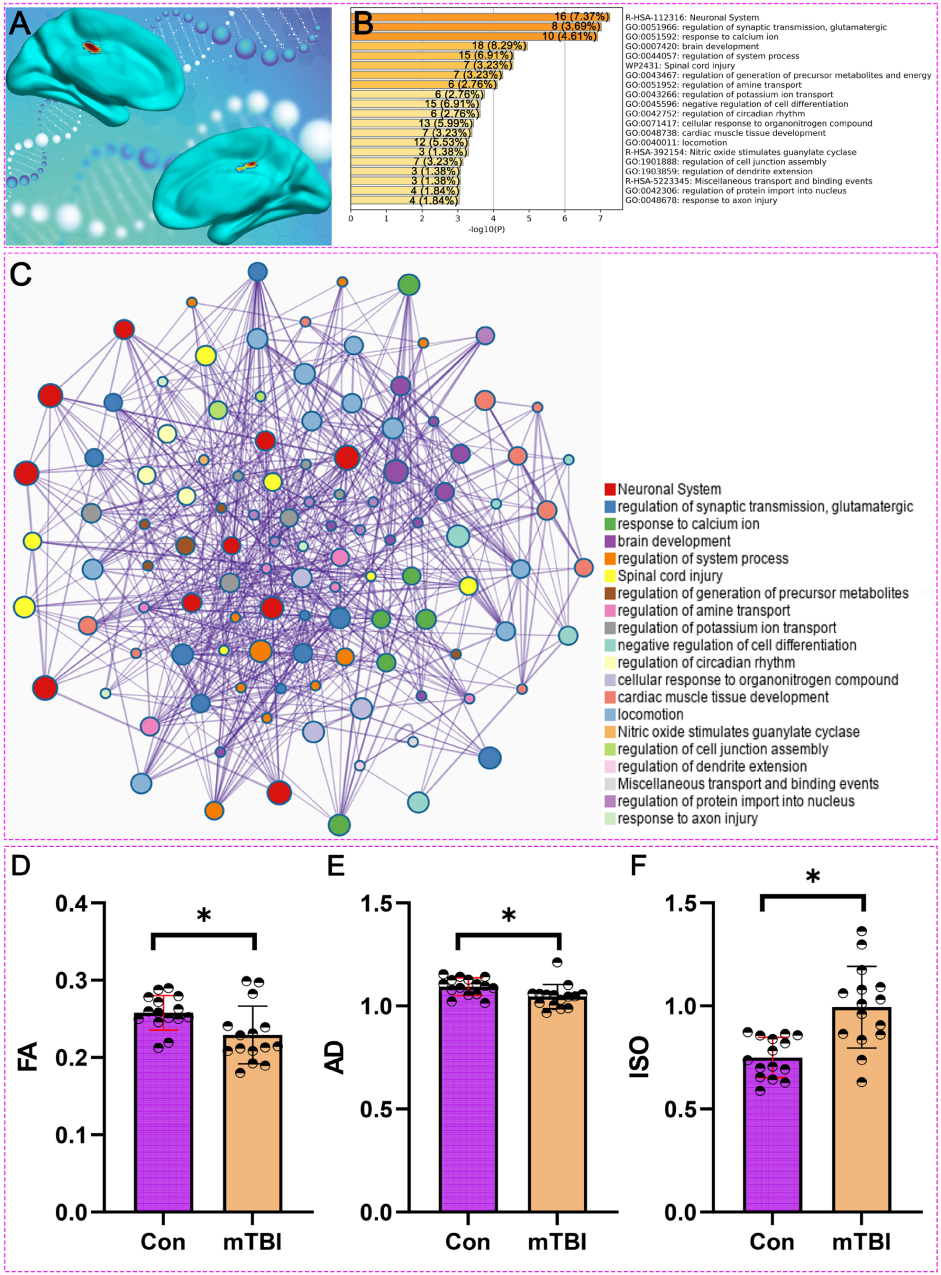
Integrated molecular and structural changes in the MCC of mTBI patients. (A) Location of the MCC: The top panel illustrates the location of the MCC as a region of interest in the brain, based on meta‐analysis findings. (B) Gene Enrichment Analysis: This panel displays the results of the gene enrichment analysis conducted on the MCC. The analysis identifies the most significantly enriched biological processes associated with the DEGs in this region, with particular emphasis on processes such as “response to axon injury”. (C) Gene Interaction Network: The comprehensive gene interaction network is depicted, where nodes represent individual genes and edges indicate the interactions between them. (D) The FA values for the MCC, comparing mTBI patients to healthy controls. Notice that a significant reduction in FA is observed in mTBI patients, indicating compromised white matter integrity in the MCC. (E) The AD values for the MCC, which reflect the magnitude of water diffusion along the principal axis of white matter fibers. Notice that in mTBI patients, AD is significantly reduced compared to healthy controls, further supporting the presence of axonal damage in the MCC. (F) The ISO values for the MCC, which measure the extent of isotropic water diffusion. Notice that in mTBI patients, ISO values are significantly higher than those in healthy controls. AD, axial diffusivity; Con, healthy control; FA, fractional anisotropy; ISO, iisotropy; mTBI, mild traumatic brain injury.

Our integrative analysis of the MCC in mTBI patients provides both molecular and structural insights into the brain's response to injury. Gene expression and enrichment analyses revealed significant upregulation of key biological processes in the MCC, including synaptic transmission, calcium ion regulation, and axon injury repair (Figure 4A–C). These molecular changes suggest compensatory mechanisms aimed at maintaining neural connectivity, particularly as the MCC has consistently shown increased DMN connectivity in mTBI patients, especially beyond 7 days post‐injury.

DTI analysis of the MCC in mTBI patients provides compelling evidence of diffuse axonal injury. We compared DTI metrics between 15 mTBI patients and 15 healthy controls to assess white matter integrity in the MCC. Baseline characteristics, including cognitive scales, were assessed in the mTBI group (Table 2). As shown in Figure 4D, FA values, which reflect the coherence of water diffusion along white matter fibers, were significantly reduced in mTBI patients compared to controls (*p* < 0.05). This reduction in FA indicates compromised white matter integrity, pointing to diffuse axonal injury in the MCC.

**TABLE 2:** Baseline characteristics including cognitive scales in 15 mTBI.

Number	Age (years)	Gender	Education (years)	Onset to image acquisition time (days)	GCS	MMSE
1	61	Male	6	7	15	26
2	36	Male	12	5	15	26
3	18	Male	12	57	15	29
4	61	Male	12	3	15	26
5	30	Male	6	2	15	23
6	68	Male	6	3	15	24
7	62	Male	12	8	15	24
8	46	Male	9	2	15	30
9	74	Male	6	8	15	22
10	62	Female	10	6	15	22
11	34	Male	12	2	15	21
12	56	Male	1	6	15	30
13	22	Female	16	4	15	29
14	58	Female	12	4	15	30
15	53	Male	10	5	15	29

Abbreviations: GCS, Glasgow Coma Score; MMSE, mini‐mental state examination.

Additionally, Figure 4E demonstrates a significant decrease in AD values in mTBI patients relative to controls (*p* < 0.05). AD measures water diffusion along the primary axis of white matter tracts, and its reduction is consistent with axonal damage, further supporting the presence of diffuse axonal injury. In Figure 4F, ISO values, which indicate the degree of isotropic water diffusion, were significantly higher in mTBI patients compared to controls (*p* < 0.05). The increase in ISO suggests alterations in the extracellular matrix or other cellular structures, reinforcing the evidence of diffuse axonal injury.

These findings support the hypothesis that diffuse axonal injury is a significant contributor to the structural and functional disruptions observed in the MCC of mTBI patients. The combination of decreased FA and AD, along with increased ISO, underscores the multifaceted nature of white matter damage in mTBI, which may underlie the connectivity changes and cognitive deficits associated with this condition.

### 3.5 Functional Enrichment Analysis of the MCC in mTBI Patients: Linking Increased Connectivity to Cognitive and Behavioral Processes

**FIGURE 5:**
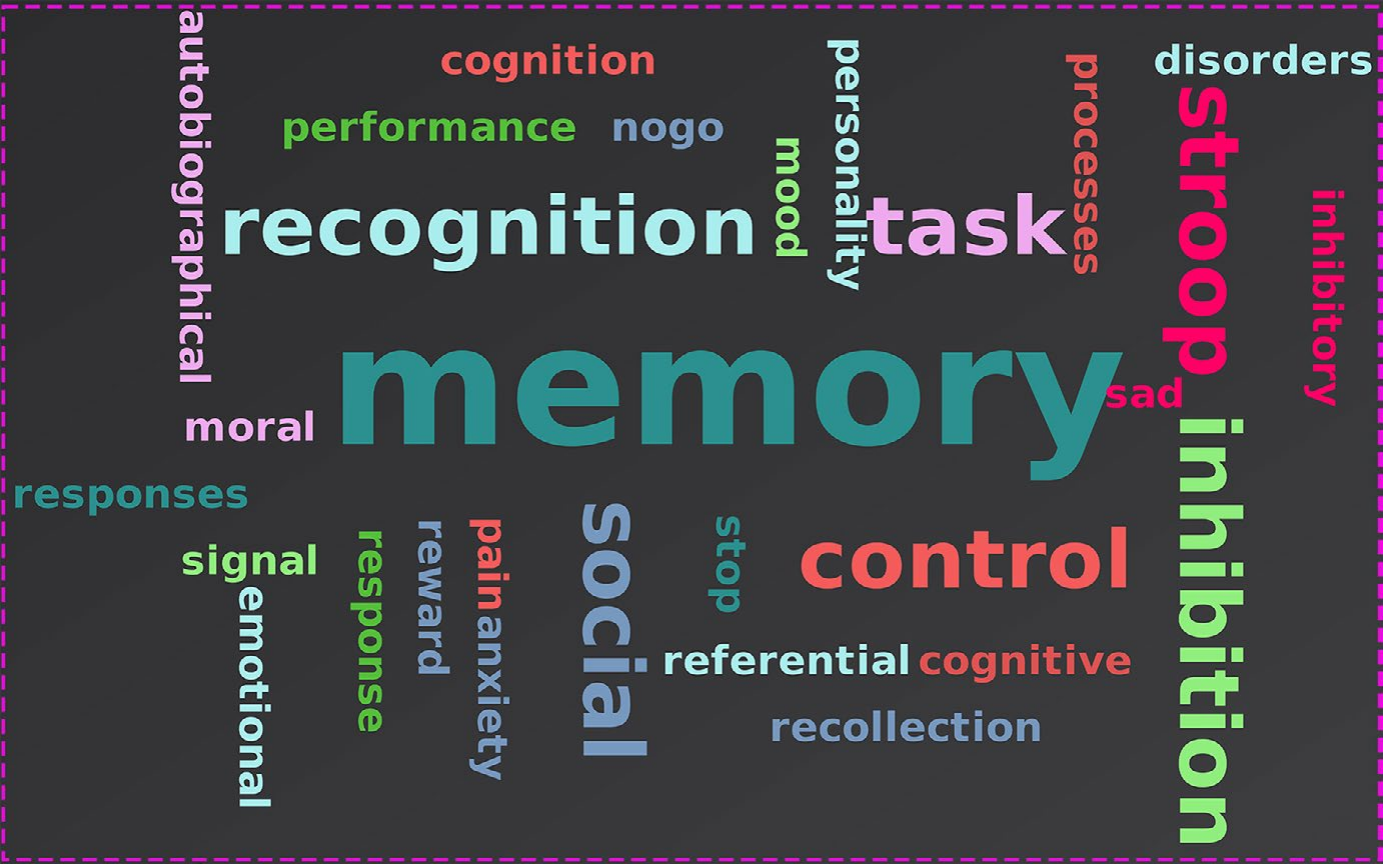
Functional enrichment analysis of the MCC as a common region of increased connectivity in mTBI patients. Key terms: Prominent terms such as “memory,” “control,” “inhibition,” “recognition,” and “social” are displayed in larger font sizes, indicating their strong relevance to the MCC's connectivity in mTBI patients. Additional relevant terms: Other notable terms, including “pain,” “anxiety,” “reward,” “cognition,” “emotional,” and “personality,” are also highlighted, reflecting the MCC's involvement in a wide range of emotional and cognitive processes. Interpretation: The word cloud effectively emphasizes the relationship between increased MCC connectivity and the diverse cognitive, emotional, and social processes affected in mTBI patients.

As shown in Figure 5, the functional enrichment analysis of the MCC—a region identified with increased connectivity in mTBI patients—highlighted significant cognitive and behavioral processes potentially affected by these neural changes. Our analysis prominently associated the MCC with processes such as “memory,” “control,” and “inhibition,” which are essential for daily cognitive functioning and often impaired in mTBI patients. These findings suggest that the increased connectivity in the MCC may contribute to difficulties in these cognitive domains following injury.

Moreover, the analysis revealed associations with emotional and social processes, including “pain,” “anxiety,” “emotional,” and “social” functions, indicating that changes in MCC connectivity may underlie mood disturbances, altered pain perception, and social challenges frequently reported by mTBI patients. Terms like “reward” and “personality” suggest broader behavioral impacts, possibly influencing patient well‐being and interpersonal interactions. Notably, these findings were validated through 1000 permutations, confirming their robustness. The consistency of these associations with known mTBI symptoms further supports the reliability of our conclusions.

Additionally, Table 2 provides detailed clinical data, including Glasgow Coma Scale (GCS) and Mini‐Mental State Examination (MMSE) scores for the 15 mTBI patients analyzed. These data contextualize the functional decoding of the MCC by offering insight into the cognitive and neurological status of the patients.

In summary, the functional enrichment analysis underscores the role of the MCC in mediating diverse cognitive, emotional, and social processes in mTBI patients, offering valuable insights into the mechanisms underlying the long‐term effects of mTBI on brain function.

### 3.6 Diagnostic Utility of DTI Metrics in Identifying mTBI Patients: Insights From ROC Curve Analysis

**FIGURE 6:**
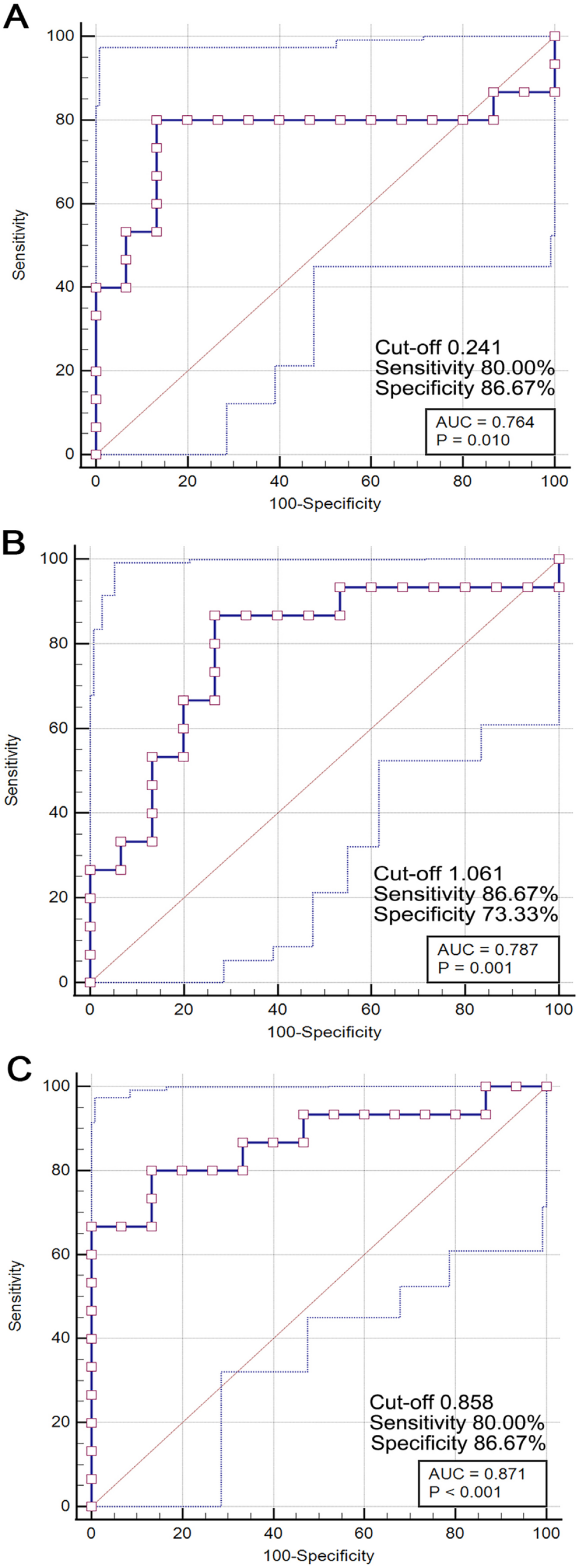
ROC curve analysis for identifying mTBI patients using DTI metrics in the MCC. (A) ROC curve for FA in the MCC. The analysis reveals that FA is a moderately effective biomarker for identifying mTBI patients. (B) ROC curve for AD in the MCC. Notice that AD is a reliable marker for distinguishing mTBI patients from healthy controls. (C) ROC curve for ISO in the MCC. Notice that ISO is a highly effective biomarker for mTBI. AUC, area under the curve.

In this study, ROC curve analysis was performed to assess the diagnostic potential of DTI metrics—FA, AD, and ISO—in identifying mTBI patients. The ROC curve for FA in the MCC revealed an AUC of 0.764, with a cut‐off value of 0.241. This resulted in a sensitivity of 80.00% and a specificity of 86.67%, indicating that FA is a moderately effective biomarker for distinguishing mTBI patients from healthy controls (Figure 6A). The analysis for AD showed an AUC of 0.787, with a cut‐off value of 1.061. The sensitivity and specificity were 86.67% and 73.33%, respectively, suggesting that AD is a reliable marker for identifying mTBI, providing slightly higher sensitivity compared to FA (Figure 6B). Additionally, the ROC curve for ISO demonstrated the highest diagnostic accuracy, with an AUC of 0.871. The optimal cut‐off value was 0.858, yielding a sensitivity of 80.00% and a specificity of 86.67%. This high AUC value indicates that ISO is a particularly strong biomarker for mTBI, especially in cases where conventional imaging fails to detect structural abnormalities (Figure 6C).

Overall, these findings highlight the potential utility of DTI metrics, particularly ISO, as valuable diagnostic tools for identifying mTBI patients, even when traditional imaging techniques may not reveal visible brain changes. This supports the integration of advanced neuroimaging techniques in the clinical assessment and diagnosis of mTBI.

### 3.7 Targeting the DLPFC for Neuromodulation in mTBI: Functional and Structural Connectivity Insights From MCC‐Based Analysis

**FIGURE 7:**
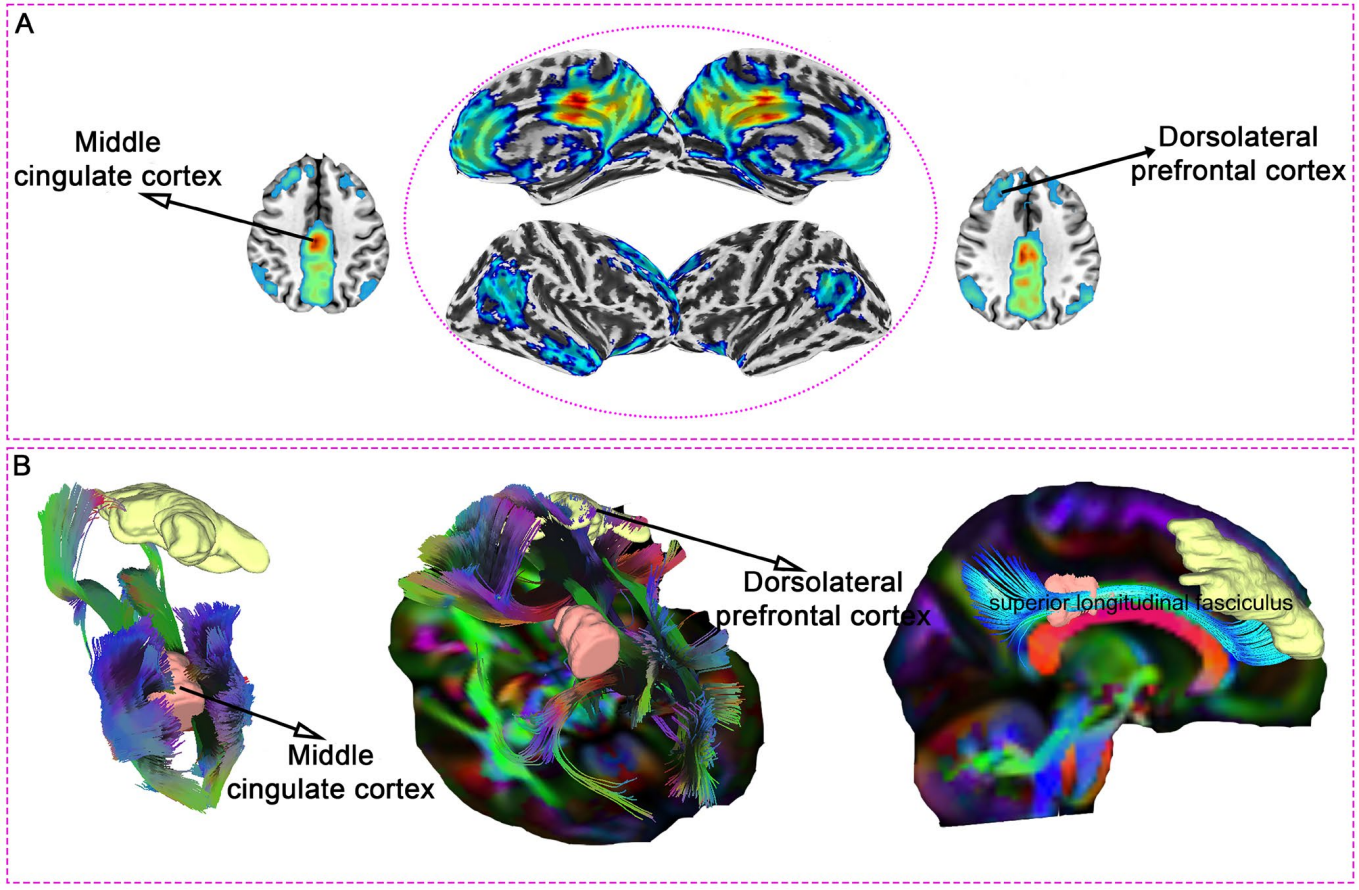
Integrated functional and structural connectivity analysis between the MCC and dorsolateral prefrontal cortex (DLPFC). (A)Whole‐brain FC analysis using the MCC as the ROI in a cohort of 40 healthy controls. Central Panel: The central 3D cortical surface visualization shows the MCC's FC with the rest of the brain. Warm colors (yellow to red) indicate regions of strong positive connectivity with the MCC, while cooler colors (blue) represent areas of weaker connectivity. Surrounding Axial Slices: The axial slices surrounding the central panel provide a more detailed view of specific brain regions with significant FC to the MCC. (B) White matter tractography analysis in mTBI patients. The left panel visualizes the white matter fiber tracts connecting the MCC and DLPFC in mTBI patients, as identified through DTI tractography. The central panel provides a close‐up of these tracts, while the right panel highlights the role of the superior longitudinal fasciculus, a major white matter pathway mediating this structural connection.

Our combined analysis of functional and structural connectivity highlights the DLPFC as a promising target for neuromodulation in mTBI. Whole‐brain FC analysis in 40 healthy controls, using the MCC as the region of interest, revealed strong connectivity between the MCC and the right DLPFC—an area critically involved in executive function and emotional regulation (Figure 7A). This robust connectivity suggests that neuromodulation techniques, such as transcranial magnetic stimulation (TMS) or transcranial direct current stimulation (tDCS), targeting the DLPFC could indirectly modulate MCC activity, potentially alleviating cognitive and emotional symptoms in mTBI patients.

Supporting these functional findings, white matter tractography in 15 mTBI patients confirmed prominent structural connections between the MCC and DLPFC, mediated by the superior longitudinal fasciculus (Figure 7B). This anatomical link provides a structural foundation for the observed FC, further reinforcing the therapeutic potential of targeting the DLPFC in neuromodulation interventions.

Together, these results offer a comprehensive framework for developing personalized neuromodulation strategies to manage persistent cognitive and emotional disturbances in mTBI by leveraging the connectivity between the MCC and DLPFC.

## 4 Discussion

In this study, we utilized a multimodal approach, combining FC analyses, DTI, and gene expression profiling to investigate DMN abnormalities in mTBI patients. Significant changes in MCC connectivity were linked to diffuse axonal injury, with reductions in FA and AD, and an increase in ISO. Gene expression analysis identified key pathways related to synaptic transmission, ion regulation, and axonal injury, potentially driving these connectivity changes. The structural link between the MCC and the right DLPFC supports the DLPFC as a target for neuromodulation therapies. This approach deepens our understanding of mTBI pathophysiology and suggests new therapeutic strategies to address network dysfunction.

Our findings align with and extend previous research on DMN alterations in mTBI. Multiple studies have consistently reported disrupted DMN connectivity following mTBI. For instance, earlier research has shown that mTBI affects brain network dynamics, particularly impacting higher‐order cognitive networks like the DMN, as well as subcortical and sensorimotor networks. The use of hidden Markov models (HMM) has further illuminated these disruptions, revealing distinct patterns in state transitions, including fractional occupancy, lifetime, and interval time [27]. Moreover, the observed differences in switching rates and transition probabilities between mTBI patients and those with cognitive impairment highlight the heterogeneity of mTBI's effects on brain function. Additionally, research has shown that an increased volume of white matter hyperintensities (WMH) in the frontal lobe is associated with reduced functional connectivity within the DMN in acute mTBI patients [28]. This reduction in connectivity, particularly in regions such as the superior frontal gyrus and anterior cingulate cortex, aligns with previous findings linking WMH to cognitive decline and disrupted network integrity. Additionally, studies have demonstrated that damage to hub regions of brain networks, including the DMN and frontoparietal network (FPN), can result in significant cognitive and behavioral impairments following mTBI [29]. In the study, researchers observed a reorganization of functional network hubs in mTBI patients, characterized by decreased participation coefficients (PC) in key networks such as the DMN and frontoparietal network (FPN). This reorganization was correlated with impaired cognitive processing and increased post‐concussion symptoms. Other studies have highlighted the crucial role of the triple network model, which includes the DMN, salience network (SN), and central executive network (CEN), in cognitive function [30]. mTBI patients exhibited significant dysregulation in the dynamic FC within these networks, with prolonged occupancy in states characterized by altered DMN and SN connectivity associated with cognitive impairment. These findings emphasize the importance of dynamic network analysis in understanding the cognitive deficits observed in mTBI patients.

Additionally, mTBI patients showed significantly increased and more variable dynamic interactions between the SN, CEN, and DMN [31]. These abnormal interactions, particularly enhanced coupling between the SN and CEN, along with reduced SN‐DMN connectivity, were associated with more severe cognitive impairments, underscoring the relevance of network dynamics in understanding TBI outcomes. Studies have also reported that mTBI disrupts functional network connectivity (FNC), impacting cognitive function [32]. Both static and dynamic FNC aberrations were observed in acute mTBI patients, particularly involving networks such as the DMN, ventral attention network (vAN), and SN, further linking these disruptions to cognitive impairments. In another study, concussion was found to increase intra‐DMN connectivity and decrease connectivity between DMN hubs and other networks, such as the visual network [33]. These connectivity changes were strongly associated with self‐reported symptoms and cognitive impairments, particularly in visual memory, highlighting the role of hub disruptions in post‐concussion cognitive and symptomatic outcomes. Furthermore, structural remodeling in the DMN, particularly in the dorsal anterior cingulate cortex (dACC) and dorsal posterior cingulate cortex (dPCC), has been implicated in the transition from acute to chronic post‐traumatic headache following mTBI. Elevated serum levels of C‐C motif chemokine ligand 2 were associated with increased dPCC volume, suggesting that inflammation plays a key role in pain modulation [3]. These findings underscore the importance of neuroinflammatory processes in driving structural changes within the DMN, potentially contributing to persistent headaches and altered pain perception in mTBI patients.

However, most previous research has primarily focused on functional alterations without thoroughly examining the structural and molecular correlates. Our study expands on this body of work by providing direct evidence of diffuse axonal injury in the MCC through DTI metrics and identifying gene expression changes that may underlie the observed FC disruptions. This integrative approach offers a more comprehensive understanding of the neurobiological impact of mTBI on brain networks.

Based on our findings, we propose the DLPFC as a potential target for neuromodulation in mTBI patients [34]. The structural connectivity between the MCC and DLPFC, revealed by white matter tractography, supports the feasibility of targeting the DLPFC to indirectly modulate MCC activity. This aligns with previous studies exploring the therapeutic potential of transcranial magnetic stimulation (TMS) and transcranial direct current stimulation (tDCS) in mTBI [35]. For instance, studies have shown that repetitive TMS (rTMS) can modulate brain connectivity, particularly within networks involved in emotional processing [36]. In one study, researchers observed changes in effective connectivity, particularly in the dACC, following rTMS treatment, suggesting that rTMS may enhance emotional health in mTBI patients by altering connectivity in brain regions involved in emotional regulation. Other studies have reported that TMS has shown promising results in alleviating depression and headaches in mTBI patients. Although tDCS has demonstrated some effectiveness, particularly for cognitive impairments, its outcomes have been more variable [37]. While these neuromodulation techniques offer valuable therapeutic options, further research is needed to optimize treatment protocols.

The DLPFC plays a critical role in cognitive functions that are often impaired following mTBI. Researchers have suggested that targeting the DLPFC with non‐invasive brain stimulation, such as intermittent theta burst stimulation (iTBS), could modulate neurophysiological activity and aid in recovery [38]. However, the effects of iTBS on the DLPFC in mTBI remain inconsistent, emphasizing the need for further research to optimize stimulation protocols and explore its therapeutic potential. Additionally, studies have demonstrated that rTMS significantly enhances activity in the DLPFC, hippocampus, and orbitofrontal cortex in patients with major depressive disorder, particularly linking hippocampal activation to symptom relief. The orbitofrontal‐hippocampal pathway has been identified as a key mediator of depression relief, suggesting its potential as a novel target for brain stimulation therapies [39]. These findings underscore the promise of DLPFC‐targeted interventions for addressing the cognitive and emotional disturbances commonly observed in mTBI.

Our study has several limitations. First, the absence of precise onset time data prevented us from stratifying patients based on the time since injury, limiting our ability to assess the progression of neural changes post‐mTBI. Future studies should adopt longitudinal designs with well‐defined onset times to better capture the evolution of neural alterations. Second, while we identified the DLPFC as a potential neuromodulation target, we did not conduct intervention studies to validate the efficacy of DLPFC‐targeted therapies in the mTBI population. Future research should focus on testing the therapeutic potential of DLPFC neuromodulation through clinical trials and exploring the molecular pathways mediating the observed connectivity changes. Additionally, longitudinal studies are necessary to assess how these neural alterations evolve over time and their relationship to long‐term outcomes in mTBI patients.

## 5 Conclusion

In conclusion, our study provides compelling evidence that diffuse axonal injury significantly contributes to DMN connectivity abnormalities in mTBI. By integrating FC analyses, DTI, and gene expression profiling, we identified the MCC as a key region of increased connectivity and axonal damage in mTBI. Moreover, the structural connectivity between the MCC and DLPFC suggests that the DLPFC could serve as a promising target for neuromodulation therapies aimed at mitigating the cognitive and emotional symptoms associated with mTBI. While our findings offer valuable insights into the neurobiological underpinnings of mTBI, further research is needed to validate the therapeutic efficacy of targeting the DLPFC and to explore the long‐term implications of these neural alterations.

## Conflicts of Interest

The authors declare no conflicts of interest.

## Supporting information


**Table S1.** The results of DMN for patients with mTBI within 7 days.
**Table S2.** The findings of DMN for patients with mTBI after 7 days.
**Table S3.** Altered DMN in patients with mTBI.

## Data Availability

The data supporting the findings of this study can be obtained from the corresponding author upon reasonable request.

## References

[1] K. Visser , M. Koggel , J. Blaauw , H. J. van der Horn , B. Jacobs , and J. van der Naalt , “Blood‐Based Biomarkers of Inflammation in Mild Traumatic Brain Injury: A Systematic Review,” Neuroscience and Biobehavioral Reviews 132 (2022): 154–168. 34826510 10.1016/j.neubiorev.2021.11.036

[2] A. Kahriman , J. Bouley , I. Tuncali , et al., “Repeated Mild Traumatic Brain Injury Triggers Pathology in Asymptomatic C9ORF72 Transgenic Mice,” Brain 146, no. 12 (2023): 5139–5152. 37527465 10.1093/brain/awad264 PMC11046056

[3] X. Niu , L. Bai , Y. Sun , et al., “Mild Traumatic Brain Injury is Associated With Effect of Inflammation on Structural Changes of Default Mode Network in Those Developing Chronic Pain,” Journal of Headache and Pain 21, no. 1 (2020): 135. 33228537 10.1186/s10194-020-01201-7 PMC7684719

[4] Y. Zhou , M. P. Milham , Y. W. Lui , et al., “Default‐Mode Network Disruption in Mild Traumatic Brain Injury,” Radiology 265, no. 3 (2012): 882–892. 23175546 10.1148/radiol.12120748 PMC3504316

[5] F. Li , L. Lu , S. Sa , et al., “Disrupted Functional Network Connectivity Predicts Cognitive Impairment After Acute Mild Traumatic Brain Injury,” CNS Neuroscience & Therapeutics 26, no. 10 (2020): 1083–1091. 32588522 10.1111/cns.13430 PMC7539836

[6] C. Sun , L. Qi , Y. Cheng , Y. Zhao , and C. Gu , “Immediate Induction of Varicosities by Transverse Compression but Not Uniaxial Stretch in Axon Mechanosensation,” Acta Neuropathologica Communications 10, no. 1 (2022): 7. 35074017 10.1186/s40478-022-01309-8 PMC8785443

[7] X. Niu , L. Bai , Y. Sun , et al., “Disruption of Periaqueductal Grey‐Default Mode Network Functional Connectivity Predicts Persistent Post‐Traumatic Headache in Mild Traumatic Brain Injury,” Journal of Neurology, Neurosurgery, and Psychiatry 90, no. 3 (2019): 326–332. 30554137 10.1136/jnnp-2018-318886

[8] F. Li , L. Lu , H. Li , et al., “Disrupted Resting‐State Functional Connectivity and Network Topology in Mild Traumatic Brain Injury: An Arterial Spin Labelling Study,” Brain Communications 5, no. 5 (2023): fcad254. 37829696 10.1093/braincomms/fcad254 PMC10567062

[9] Y. Sun , S. Wang , S. Gan , et al., “Serum Neuron‐Specific Enolase Levels Associated With Connectivity Alterations in Anterior Default Mode Network After Mild Traumatic Brain Injury,” Journal of Neurotrauma 38, no. 11 (2021): 1495–1505. 33687275 10.1089/neu.2020.7372

[10] F. Li , D. Zhang , J. Ren , et al., “Connectivity of the Insular Subdivisions Differentiates Posttraumatic Headache‐Associated From Nonheadache‐Associated Mild Traumatic Brain Injury: An Arterial Spin Labelling Study,” Journal of Headache and Pain 25, no. 1 (2024): 103. 38898386 10.1186/s10194-024-01809-z PMC11186101

[11] T. Shao , J. Huang , Y. Zhao , et al., “Metformin Improves Cognitive Impairment in Patients With Schizophrenia: Associated With Enhanced Functional Connectivity of Dorsolateral Prefrontal Cortex,” Translational Psychiatry 13, no. 1 (2023): 315. 37821461 10.1038/s41398-023-02616-x PMC10567690

[12] A. Liberati , D. G. Altman , J. Tetzlaff , et al., “The PRISMA Statement for Reporting Systematic Reviews and Meta‐Analyses of Studies That Evaluate Healthcare Interventions: Explanation and Elaboration,” BMJ 339 (2009): b2700. 19622552 10.1136/bmj.b2700 PMC2714672

[13] T. Madsen , A. Erlangsen , S. Orlovska , R. Mofaddy , M. Nordentoft , and M. E. Benros , “Association Between Traumatic Brain Injury and Risk of Suicide,” Journal of the American Medical Association 320, no. 6 (2018): 580–588. 30120477 10.1001/jama.2018.10211 PMC6142987

[14] N. D. Silverberg and G. L. Iverson , “Expert Panel Survey to Update the American Congress of Rehabilitation Medicine Definition of Mild Traumatic Brain Injury,” Archives of Physical Medicine and Rehabilitation 102, no. 1 (2021): 76–86. 33035515 10.1016/j.apmr.2020.08.022

[15] A. Stang , “Critical Evaluation of the Newcastle‐Ottawa Scale for the Assessment of the Quality of Nonrandomized Studies in Meta‐Analyses,” European Journal of Epidemiology 25, no. 9 (2010): 603–605. 20652370 10.1007/s10654-010-9491-z

[16] J. L. Lancaster , D. Tordesillas‐Gutiérrez , M. Martinez , et al., “Bias Between MNI and Talairach Coordinates Analyzed Using the ICBM‐152 Brain Template,” Human Brain Mapping 28, no. 11 (2007): 1194–1205. 17266101 10.1002/hbm.20345 PMC6871323

[17] E. H. Shen , C. C. Overly , and A. R. Jones , “The Allen Human Brain Atlas: Comprehensive Gene Expression Mapping of the Human Brain,” Trends in Neurosciences 35, no. 12 (2012): 711–714. 23041053 10.1016/j.tins.2012.09.005

[18] Y. Zhou , B. Zhou , L. Pache , et al., “Metascape Provides a Biologist‐Oriented Resource for the Analysis of Systems‐Level Datasets,” Nature Communications 10, no. 1 (2019): 1523. 10.1038/s41467-019-09234-6 PMC6447622 30944313

[19] C. Sheth , J. Rogowska , M. Legarreta , E. McGlade , and D. Yurgelun‐Todd , “Functional Connectivity of the Anterior Cingulate Cortex in Veterans With Mild Traumatic Brain Injury,” Behavioural Brain Research 396 (2021): 112882. 32853657 10.1016/j.bbr.2020.112882

[20] J. Amir , J. K. R. Nair , R. Del Carpio‐O'Donovan , et al., “Atypical Resting State Functional Connectivity in Mild Traumatic Brain Injury,” Brain and Behavior: A Cognitive Neuroscience Perspective 11, no. 8 (2021): e2261. 10.1002/brb3.2261 PMC8413771 34152089

[21] M. M. D'Souza , M. Kumar , A. Choudhary , et al., “Alterations of Connectivity Patterns in Functional Brain Networks in Patients With Mild Traumatic Brain Injury: A Longitudinal Resting‐State Functional Magnetic Resonance Imaging Study,” Neuroradiology Journal 33, no. 2 (2020): 186–197. 31992126 10.1177/1971400920901706 PMC7140304

[22] D. L. Murdaugh , T. Z. King , B. Sun , et al., “Longitudinal Changes in Resting State Connectivity and White Matter Integrity in Adolescents With Sports‐Related Concussion,” Journal of the International Neuropsychological Society 24, no. 8 (2018): 781–792. 30139405 10.1017/S1355617718000413

[23] V. M. Vergara , A. R. Mayer , E. Damaraju , K. A. Kiehl , and V. Calhoun , “Detection of Mild Traumatic Brain Injury by Machine Learning Classification Using Resting State Functional Network Connectivity and Fractional Anisotropy,” Journal of Neurotrauma 34, no. 5 (2017): 1045–1053. 27676221 10.1089/neu.2016.4526 PMC5333571

[24] D. E. Nathan , T. R. Oakes , P. H. Yeh , et al., “Exploring Variations in Functional Connectivity of the Resting State Default Mode Network in Mild Traumatic Brain Injury,” Brain Connectivity 5, no. 2 (2015): 102–114. 25222050 10.1089/brain.2014.0273

[25] C. Sours , J. Zhuo , S. Roys , K. Shanmuganathan , and R. P. Gullapalli , “Disruptions in Resting State Functional Connectivity and Cerebral Blood Flow in Mild Traumatic Brain Injury Patients,” PLoS One 10, no. 8 (2015): e0134019. 26241476 10.1371/journal.pone.0134019 PMC4524606

[26] M. C. Stevens , D. Lovejoy , J. Kim , H. Oakes , I. Kureshi , and S. T. Witt , “Multiple Resting State Network Functional Connectivity Abnormalities in Mild Traumatic Brain Injury,” Brain Imaging and Behavior 6, no. 2 (2012): 293–318. 22555821 10.1007/s11682-012-9157-4

[27] L. Lu , F. Li , H. Li , L. Zhou , X. Wu , and F. Yuan , “Aberrant Dynamic Properties of Whole‐Brain Functional Connectivity in Acute Mild Traumatic Brain Injury Revealed by Hidden Markov Models,” CNS Neuroscience & Therapeutics 30, no. 3 (2024): e14660. 38439697 10.1111/cns.14660 PMC10912843

[28] D. Zhang , P. Zhu , B. Yin , et al., “Frontal White Matter Hyperintensities Effect on Default Mode Network Connectivity in Acute Mild Traumatic Brain Injury,” Frontiers in Aging Neuroscience 13 (2021): 793491. 35250532 10.3389/fnagi.2021.793491 PMC8890121

[29] L. Bai , B. Yin , S. Lei , et al., “Reorganized Hubs of Brain Functional Networks After Acute Mild Traumatic Brain Injury,” Journal of Neurotrauma 40, no. 1–2 (2023): 63–73. 35747994 10.1089/neu.2021.0450

[30] H. Liu , G. Zhang , H. Zheng , et al., “Dynamic Dysregulation of the Triple Network of the Brain in Mild Traumatic Brain Injury and Its Relationship With Cognitive Performance,” Journal of Neurotrauma 41, no. 7–8 (2024): 879–886. 37128187 10.1089/neu.2022.0257

[31] X. Li , X. Jia , Y. Liu , et al., “Brain Dynamics in Triple‐Network Interactions and Its Relation to Multiple Cognitive Impairments in Mild Traumatic Brain Injury,” Cerebral Cortex 33, no. 11 (2023): 6620–6632. 36610729 10.1093/cercor/bhac529

[32] L. Lu , J. Zhang , F. Li , et al., “Aberrant Static and Dynamic Functional Network Connectivity in Acute Mild Traumatic Brain Injury With Cognitive Impairment,” Clinical Neuroradiology 32, no. 1 (2022): 205–214. 34463779 10.1007/s00062-021-01082-6

[33] H. C. Bouchard , K. L. Higgins , G. K. Amadon , et al., “Concussion‐Related Disruptions to Hub Connectivity in the Default Mode Network Are Related to Symptoms and Cognition,” Journal of Neurotrauma 41, no. 5–6 (2024): 571–586. 37974423 10.1089/neu.2023.0089

[34] M. J. Cho , H. D. Lee , J. W. Kim , and S. H. Jang , “Relationship Between Short‐Term Memory Impairment and the Dorsolateral Prefrontal Cortex Injury in Patients With Mild Traumatic Brain Injury,” Journal of Integrative Neuroscience 21, no. 3 (2022): 93. 35633174 10.31083/j.jin2103093

[35] N. S. Philip , D. Ramanathan , B. Gamboa , et al., “Repetitive Transcranial Magnetic Stimulation for Depression and Posttraumatic Stress Disorder in Veterans With Mild Traumatic Brain Injury,” Neuromodulation 26, no. 4 (2023): 878–884. 36737300 10.1016/j.neurom.2022.11.015 PMC10765323

[36] T. Sultana , M. A. Hasan , X. Kang , V. Liou‐Johnson , M. M. Adamson , and A. Razi , “Neural Mechanisms of Emotional Health in Traumatic Brain Injury Patients Undergoing rTMS Treatment,” Molecular Psychiatry 28, no. 12 (2023): 5150–5158. 37414927 10.1038/s41380-023-02159-z PMC11041778

[37] A. Mollica , R. Greben , C. Oriuwa , S. H. Siddiqi , and M. J. Burke , “Neuromodulation Treatments for Mild Traumatic Brain Injury and Post‐Concussive Symptoms,” Current Neurology and Neuroscience Reports 22, no. 3 (2022): 171–181. 35175543 10.1007/s11910-022-01183-w

[38] H. L. Coyle , N. W. Bailey , J. Ponsford , and K. E. Hoy , “Investigation of Neurobiological Responses to Theta Burst Stimulation During Recovery From Mild Traumatic Brain Injury (mTBI),” Behavioural Brain Research 442 (2023): 114308. 36702385 10.1016/j.bbr.2023.114308

[39] S. Han , X.‐X. Li , S. Wei , et al., “Orbitofrontal Cortex‐Hippocampus Potentiation Mediates Relief for Depression: A Randomized Double‐Blind Trial and TMS‐EEG Study,” Cell Reports Medicine 4, no. 6 (2023): 101060. 37263267 10.1016/j.xcrm.2023.101060 PMC10313932

