# Supplementary material for: Clinical Insights Into Default Mode Network Abnormalities in Mild Traumatic Brain Injury: Unraveling Axonal Injury Through Functional, Structural, and Molecular Analyses
Source: CNS Neurosci Ther. 2024 Dec 26;30(12):e70188. doi: 10.1111/cns.70188 (PMC11669841; doi:10.1111/cns.70188)
Supplement: Supplementary file 1 — Table S1. The results of DMN for patients with mTBI within 7 days. Table S2. The findings of DMN for patients with mTBI after 7 days. Table S3. Altered DMN in patients with mTBI. [file CNS-30-e70188-s001.docx]

Supplementary Table S1. The results of DMN for patients with mTBI within 7 days.

| **Cluster** | **Regions** | **MNI**  **x y z** | **ALE (×10^-3^)** | **Z value** | ***P* (×10^-4^)** |
| --- | --- | --- | --- | --- | --- |
| **mTBI > Health controls** | | | | | |
| 1 | Posterior Lobe, Declive | -30, -66, -20 | 2.73 | 3.55 | 1.92 |
| 2 | Temporal Lobe, Superior Temporal Gyrus | -50, -22, -2 | 3.22 | 4.21 | 1.25 |
| 3 | Parietal Lobe, Precuneus | -26, -68, 38 | 2.73 | 3.55 | 1.92 |
| 4 | Anterior Lobe, Culmen | 0, -62, -19 | 3.08 | 3.92 | 4.48 |
| **mTBI < Health controls** | | | | | |
| 1 | Parietal Lobe, Angular Gyrus | 42, -54, 38 | 1.00 | 2.33 | 97.50 |

MNI: Montreal Neurological Institute; ALE: Activation likelihood estimation;

mTBI: Mild traumatic brain injury

Supplementary Table S2. The findings of DMN for patients with mTBI after 7 days.

| **Cluster** | **Regions** | **MNI**  **x y z** | **ALE (×10^-3^)** | **Z value** | ***P* (×10^-4^)** |
| --- | --- | --- | --- | --- | --- |
| **mTBI > Health controls** | | | | | |
| 1 | Limbic Lobe, Cingulate Gyrus | -8, -24, 36 | 3.38 | 3.70 | 1.06 |
| 2 | Limbic Lobe, Cingulate Gyrus | 6, -26, 38 | 3.24 | 3.64 | 1.37 |
| 3 | Brodmann area 23 | 0, -16, 34 | 3.19 | 3.61 | 1.52 |
| 4 | Insula, Brodmann area 13 | 46, 4, 2 | 3.15 | 3.59 | 1.66 |
| 5 | Claustrum | 38, -6, 4 | 2.84 | 3.27 | 5.38 |
| 6 | Postcentral Gyrus, Brodmann area 7 | -6, -52, 72 | 3.18 | 3.61 | 1.55 |
| 7 | Postcentral Gyrus, Brodmann area 7 | 10, -52, 74 | 3.03 | 3.48 | 2.54 |
| 8 | Superior Frontal Gyrus, Brodmann area 9 | 30, 54, 28 | 3.14 | 3.56 | 1.87 |
| 9 | Superior Frontal Gyrus, Brodmann area 9 | 24, 56, 28 | 3.09 | 3.50 | 2.30 |

MNI: Montreal Neurological Institute; ALE: Activation likelihood estimation;

mTBI: Mild traumatic brain injury

Supplementary Table S3. Altered DMN in patients with mTBI.

| **Cluster** | **Regions** | **MNI**  **x y z** | **ALE (×10^-3^)** | **Z value** | ***P* (×10^-4^)** |
| --- | --- | --- | --- | --- | --- |
| **mTBI > Health controls** | |  |  |  |  |
| 1 | Limbic Lobe, Cingulate Gyrus | -8, -24, 36 | 3.38 | 3.47 | 2.58 |
| 2 | Limbic Lobe, Cingulate Gyrus | 6, -26, 38 | 3.24 | 3.40 | 3.33 |
| 3 | Limbic Lobe, Cingulate Gyrus | 0, -16, 34 | 3.19 | 3.37 | 3.70 |
| **mTBI < Health controls** | |  |  |  |  |
| 1 | Limbic Lobe, Posterior Cingulate | 14, -60, 16 | 3.07 | 3.51 | 2.22 |
| 2 | Occipital Lobe, Precuneus | 4, -68, 22 | 2.81 | 3.17 | 7.71 |

MNI: Montreal Neurological Institute; ALE: Activation likelihood estimation;

mTBI: Mild traumatic brain injury
